# Metabolomic and Transcriptomic Analyses Reveal That a MADS-Box Transcription Factor *TDR4* Regulates Tomato Fruit Quality

**DOI:** 10.3389/fpls.2019.00792

**Published:** 2019-06-19

**Authors:** Xiaodan Zhao, Xinyu Yuan, Sha Chen, Da-Qi Fu, Cai-Zhong Jiang

**Affiliations:** ^1^School of Food and Chemical Engineering, Beijing Technology and Business University, Beijing, China; ^2^Laboratory of Food Biotechnology, College of Food Science and Nutritional Engineering, China Agricultural University, Beijing, China; ^3^Institute of Traditional Chinese Medicine, China Academy of Chinese Medical Sciences, Beijing, China; ^4^Department of Plant Sciences, University of California, Davis, Davis, CA, United States; ^5^Crops Pathology and Genetics Research Unit, United States Department of Agriculture, Agricultural Research Service, Davis, CA, United States

**Keywords:** transcription factors, virus-induced gene silencing, *TDR4*, fruit quality, metabolomic, transcriptomic

## Abstract

Tomato fruit ripening is a complex process, which determines the formation of fruit quality. Many factors affect fruit ripening, including environmental conditions and genetic factors. Transcription factors (TFs) play key roles in regulating fruit ripening and quality formation. Current studies have found that the *TDR4* gene is an important TF for tomato fruit ripening, but its effects on fruit metabolism and quality are less well studied. In this study, suppression of *TDR4* gene expression obtained through virus-induced gene silencing (VIGS) technology resulted in an orange pericarp phenotype. Transcriptomic analysis of *TDR4*-silenced fruit showed changes in the expression of genes involved in various metabolic pathways, including amino acid and flavonoid biosynthesis pathways. Metabolomic analysis showed that levels of several amino acids including phenylalanine and tyrosine, and organic acids were reduced in *TDR4*-silenced fruit, while α-tomatine accumulated in *TDR4*-silenced fruit. Taken together, our RNA-seq and metabolomics analyses of *TDR4*-silenced fruit showed that *TDR4* is involved in ripening and nutrient synthesis in tomato fruit, and is therefore an important regulator of fruit quality.

## Introduction

Fruit is an important source of human healthy diet which can provide vitamins, minerals, and a wide range of bioactive compounds, including antioxidant carotenoids and various polyphenols (Seymour et al., 2013). The quality and nutrition of fresh fruits are gradually formed during ripening. Studying the molecular mechanism of fruit ripening is an important way to understand the formation of fruit quality. Fruit ripening is a complex biological process to form delicious and nutritious fruits for attracting animals to eat and spread seeds (Martel et al., 2011). Some general ripening-associated changes take place among some fruit species, including the cell wall degradation for fruit softening, alteration of the composition and levels of secondary metabolites, such as pigments, flavors, and aromas during fruit ripening (Martel et al., 2011). These changes are influenced by multiple genetic and biochemical pathways that are regulated by several critical transcription factors (TFs) (Giovannoni, 2007).

The tomato (*Solanum lycopersicum*) is the main horticultural crops and it is hot popular food for consumers. Tomato is considered as an ideal model material for studying fleshy fruit ripening (Klee and Giovannoni, 2011; Karlova et al., 2014). In climacteric fruits, including tomatoes, increased ethylene production is required for the onset of ripening (Barry and Giovannoni, 2007). During fruit development and ripening, the biosynthesis and signal transduction of ethylene are both regulated by several TFs, including RIPENING INHIBITOR (MADS-RIN), COLORLESS NON-RIPENING (CNR), NON-RIPENING (NOR), TOMATO AGAMOUS-LIKE1 (TAGL1), NOR-like1, and APETALA2a (AP2a) (Vrebalov et al., 2002, 2009; Manning et al., 2006; Giovannoni, 2007; Karlova et al., 2011; Gao et al., 2018b, 2019). TDR4/FUL1 and its homolog MBP7/FUL2 are MADS-box family TFs with high sequence similarity to *Arabidopsis* FRUITFULL. In contrast to the above-mentioned TFs, TDR4/FUL1 and MBP7/FUL2 do not regulate ethylene biosynthesis but affect fruit ripening in an ethylene-independent manner (Bemer et al., 2012). A previous study revealed that TDR4/FUL1 mRNA and protein accumulate during ripening in tomato fruit, while MBP7/FUL2 mRNA and protein accumulate during the pre-ripening stage and throughout ripening process (Shima et al., 2013). RNAi-silencing of each of the *FUL* homologs independently results in very mild changes to tomato fruit pigmentation, while the silencing of both genes results in an orange ripe fruit with highly reduced levels of lycopene, suggesting that FUL1/TDR4 and FUL2/MBP7 possess redundant functions in fruit ripening (Bemer et al., 2012). The expression of genes involved in cell wall modification, cuticle production, volatile production, and glutamate accumulation was also altered in *TDR4* silencing tomato fruit (Bemer et al., 2012). Chromatin immunoprecipitation coupled with microarray analysis (ChIP-chip) revealed that FUL homologs take part in many biological processes through the regulation of ripening-related gene expression, both in cooperation with and independent of RIN (Fujisawa et al., 2014).

In order to further study the effect of TDR4 on tomato quality metabolism, we utilized virus-induced gene silencing (VIGS) to silence *TDR4* in tomato fruit. Analysis of transcripts and metabolites of *TDR4*-silened fruit indicated that it was involved in the metabolism of several amino acids and biosynthesis of secondary metabolites, altering fruit nutrient levels and flavor. The result shows that *TDR4* regulates the nutrient levels and quality of tomato fruit.

## Materials and Methods

### Plant Material and Growth Conditions

Tomato plants (*S. lycopersicum* “Ailsa Craig”) were planted in commercial tomato-cultivated soil and grown under standard glasshouse conditions of 16-h day length and 25°C, with a night temperature of 18°C with 75% relative humidity. Flowers were tagged at 1 day post-anthesis (DPA). Ten plants are for control and 10 plants were used to silence *TDR4* gene; each plant was no less than 15 fruits.

### Vector Construction

The tobacco rattle virus (TRV)-based vectors pTRV1 and pTRV2 were used for VIGS. To construct a pTRV2-*TDR4* recombinant, a 360-bp *Eco*RI/*Bam*HI-containing DNA fragment of the *TDR4* gene, corresponding to nucleotides 323–682 (NM_001247244.2), was amplified from tomato fruit complementary DNA (cDNA) using primers *TDR4*-VIGS-For and *TDR4*-VIGS-Rev (Supplementary Table S1). The resulting products and pTRV2 vector were digested with *EcoRI/BamHI* and ligated by T4 ligase.

### Agro-Infiltration

The VIGS assay was carried out as previously described (Fu et al., 2005) with slight modification. All plant inoculations were performed using a 1:1 (v/v) mixture of two *Agrobacterium tumefaciens* GV3101 cultures, one containing the pTRV1 vector and the other containing the pTRV2 or pTRV2-derived vector. Bacterial clones were grown overnight at 28°C in Luria-Bertani medium containing 10 mM MES and 20 mM acetosyringone with kanamycin, gentamycin, and rifampicin antibiotics. They were then harvested and transferred to the infiltration medium [10 mM MgCl_2_, 10 mM MES (pH 5.6), 200 mM acetosyringone] to a final OD_600_ of 6.0. For co-infiltration studies, 1:1 mixtures of pTRV1, pTRV2-00, or pTRV2-*TDR4* were used. The *Agrobacterium* mixture was injected into the carpopodium of the tomato fruit at 7–10 DPA after pollination using a 1-ml syringe with a syringe needle. The control fruits were infected by *A. tumefaciens* containing a pTRV2 empty vector, and the *TDR4*-silenced fruits were infected by *A. tumefaciens* containing a pTRV2-TDR4 vector. Each infected fruit was not less than 100 from 10 different plants.

### RNA-Seq and Data Processing

Total RNA was extracted from the fruit pericarp of TRV2-00 infected control fruits and TRV2-*TDR4* silenced fruits (three biological replicates in which each sample was collected from six different fruits) using a RNeasy MiniKit (Qiagen, Hilden, Germany) (Wang et al., 2016). RNA integrity was evaluated on 1% agarose gels stained with ethidium bromide (EB). RNA concentrations were measured using a Nano Photometer^®^ spectrophotometer (Implen, CA, United States). cDNA libraries were generated using the NEBNext^®^ Ultra RNA Library Prep Kit for Illumina^®^ (New England Biolabs, Ipswich, MA, United States) following the manufacturer’s instructions. Briefly, mRNA was enriched using oligo (dT)-attached magnetic beads. Fragmentation was performed by divalent cations in NEBNext First Strand Synthesis Reaction Buffer. These fragments were used to synthesize first-strand cDNA using random hexamer primers and M-MuLV Reverse Transcriptase. Then, second-strand cDNA synthesis was achieved using DNA Polymerase I and RNase H. Exonuclease/polymerase activities were used to convert overhangs into blunt ends. In order to select cDNA fragments of the appropriate size, library fragments were purified with the AMPure XP system (Beckman Coulter, Beverly, MA, United States). USER Enzyme (New England Biolabs) was subsequently used with size-selected, adaptor-ligated cDNA. Then, PCR was carried out with Phusion High-Fidelity DNA polymerase, universal PCR primers, and Index (X) Primer. Finally, PCR products were purified, and library quality was evaluated on the Agilent Bioanalyzer 2100 system (Palo Alto, CA, United States). Clustering of the index-coded samples was performed on a cBot Cluster Generation System using the HiSeq 4000 PE Cluster Kit (Illumina, San Diego, CA, United States) according to the manufacturer’s instructions. Then, library preparations were sequenced on an Illumina HiSeq 4000 platform, and 150-bp paired-end reads were generated.

We used cutadapt^1^ and the FASTX-Toolkit^2^ to trim raw reads in order to remove barcode and adaptor sequences, and the resulting clean reads were checked for quality using a threshold of Q < 20. Clean reads from each library were aligned to the tomato reference genome (SGN release version SL2.50^3^) using TopHat^4^. We used Cufflinks^5^ to assemble reads with fewer than two mismatches. Differentially expressed genes (DEGs) between pTRV2-*TDR4* andpTRV2-00 were identified with the following criteria: fold-change ≥ 2 and Q-value < 0.05. Clean reads of RNA-seq were deposited in the National Center for Biotechnology Information Sequence Read Archive^6^ under accession number SRP201254.

### Gene Enrichment and Pathway Analysis

To assess the distribution of DEG functions, Gene Ontology (GO) enrichment analysis was performed using WEGO^7^. FASTA format files containing DEG cDNA sequences were obtained using Perl scripts, and then pathway analysis was conducted with the Kyoto Encyclopedia of Genes and Genomes (KEGG) in KOBAS^8^, based on native BLAST tools and organism annotation libraries.

### Validation of RNA-Seq by Quantitative Real-Time PCR(qRT-PCR)

Total RNA (2 μg) from three biological replicates was purified and reverse-transcribed into cDNA using TranScript One-step gDNA Removal and cDNA Synthesis SuperMix (TransGen Biotech, Beijing, China) with oligo(dT). Then, qRT-PCR was performed with SYBR Green PCR SuperMix (TransGen Biotech) on a Bio-Rad Real-Time PCR System CFX96 (Bio-Rad, Hercules, CA, United States), using a tomato actin gene as a reference gene. All primer sequences are listed in Supplementary Table S1. The reaction proceeded as follows: 95°C for 10 min, followed by 40 cycles of 95°C for 15 s and 60°C for 30 s. The fluorescence signal was monitored automatically at each cycle. Relative expression levels of specific mRNAs were measured using the 2^-ΔΔCt^ method. Standard errors were calculated based on a minimum of three biological replicates.

### Liquid Chromatography-Tandem Mass Spectrometry (LC-MS/MS) Analysis

Frozen TRV2-00 and TRV2-*TDR4* tomato pericarp samples (six biological replicates) were milled into powder using a mortar and pestle, and the powder from each sample was weighed. Next, 100 mg was suspended in either 1.0 ml pure methanol or 1.0 ml 75% aqueous methanol for the extraction of lipid-soluble and water-soluble metabolites, respectively. Both types of methanol contained 20 mg l^-1^ lidocaine and 20 g l^-1^ CHAPS. The suspensions were vortexed and then extracted at 4°C overnight. Following centrifugation at 12,000 × *g* for 10 min, the supernatants of both the lipid-soluble and water-soluble metabolites were collected and mixed in a ratio of 1:1 before being filtered through a 0.45-μm membrane and subjected to LC-MS analysis.

A high performance liquid chromatography unit, equipped with a photodiode array detector (HPLC-20A, Shimadzu, Japan), was used to analyze the metabolites in the tomato extract. The separation of metabolites was carried out under the following conditions: column: Eclipse XDB-C_18_ (3.0 mm × 50 mm); solvent A: water with 0.2% formic acid; solvent B: acetonitrile; gradient program: 95:5 A:B (v/v) at 0 min, 5:95 A:B at 12 min, 5:95 A:B at 15 min, 95:5 A:B at 15.1 min, 95:5 A:B at 22 min; flow rate: 0.2 ml min^-1^; temperature: 45°C; injection volume: 2 μl. The masses of the eluted compounds ranging from 50 to 1500 *m z*^-1^ were monitored with a Triple Quad LC/MS equipped with an electrospray ionization (ESI) source.

Quantitative detection was performed using an UHPLC-ESI-QQQ-MS (Agilent 1290 and 6460 triple quadrupole mass spectrometry series). An ESI source working either in positive or negative ion mode was used for all MS analyses, with nitrogen as the drying agent. The MS conditions in positive mode were as follows: HV voltage: 4000 kV; capillary: 7 μg; nozzle voltage: 500 V; delta EMV: 300 V; gas flow: 5 l min^-1^; gas temperature: 400°C; sheath gas flow: 11 l min^-1^. Collision energy was optimized based on the standards. Helium was used as the collision gas for collision-induced dissociation (CID). Quantification was performed using the multiple reaction monitoring (MRM) mode under unit mass-resolution conditions. The data were processed with MassHunter software.

## Results and Discussion

### Silencing of *TDR4* Inhibits Tomato Fruit Ripening

**FIGURE 1:**
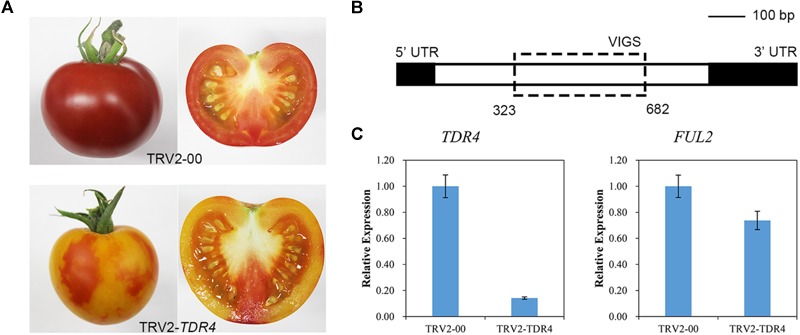
VIGS in tomato fruit. **(A)** Phenotype of *TDR4*-silenced tomato fruit. TRV2-00 was used as a control. **(B)** Schematic representation of the structure of the *TDR4* coding sequence and the positions of the fragments used for VIGS. **(C)** Silencing efficiency of *TDR4* and *FUL2* genes.

To silence *TDR4* gene and analyze its effect on tomato fruit metabolism, a mixture of *Agrobacterium* cultures containing pTRV-*TDR4* and pTRV1 was injected into the carpopodium of the tomato fruit at 7–10 days after pollination using a 1-ml syringe with a needle. Around 35 days post-Agro-injection, approximately 35 tomato fruits injected with pTRV-*TDR4* failed to turn red and developed an orange phenotype at the red ripening (RR) stage. All control fruit injected with *A. tumefaciens* containing pTRV1 and pTRV2-00 turned red normally, like the wild-type fruit (Figure 1A). Quantitative real-time PCR (qRT-PCR) was performed to confirm *TDR4* silencing. The primers that anneal to the *TDR4* gene outside the region targeted for silencing were used (Figure 1B). In pTRV-*TDR4* injected fruits, the *TDR4* message was reduced by more than 80% compared with the TRV injected controls (Figure 1C). The level of *actin* gene RNA was similar in TRV-*TDR4* and TRV alone injected tissue and served as an internal control for RNA quality and RT-qPCR. Based on the above results, we can conclude that the *TDR4* gene has been successfully silenced in tomato fruit and *TDR4*-silenced fruits can be used for subsequent studies on the effects of *TDR4* gene on tomato fruit metabolism.

### Global Overview of RNA-Seq Profile of *TDR4*-Silenced Tomato

To test the molecular consequences of silencing the *TDR4* gene in *TDR4*-silenced fruit, we compared the gene expression levels in the pericarp of *TDR4*-silenced fruit with that in control pericarp at red stage using strand-specific mRNA sequencing. The result showed that all clean reads were mapped and aligned against the tomato reference genome (ITAG2.4). Within each file, 79.1 ± 2.1% of the reads were uniquely aligned, suggesting that the sequencing results were effective and reliable (Table 1).

**Table 1:** Summary of clean read counts and percentage of unique mapped reads.

Sample	Clean reads left/right	Left unique mapped	Right unique mapped	Unique aliment
TRV2-00-1^a^	21377728 (100%)	18987592 (88.8%)	18084744 (84.6%)	17254526 (80.7%)
TRV2-00-2	23506338 (100%)	21188488 (90.1%)	19683578 (83.7%)	18860690 (80.2%)
TRV2-00-3	22897923 (100%)	20740458 (90.6%)	18994540 (83.0%)	18160445 (79.3%)
TRV2-TDR4-1	21079921 (100%)	19277894 (91.5%)	17904806 (84.9%)	17144044 (81.3%)
TRV2-TDR4-2	19561207 (100%)	17455736 (89.2%)	15730401 (80.4%)	14925877 (76.3%)
TRV2-TDR4-3	22102725 (100%)	19841339 (89.8%)	17906852 (81.0%)	17006260 (76.9%)

*^*a*^ Sample labels 1, 2, and 3 indicate three biological replicates.*

**FIGURE 2:**
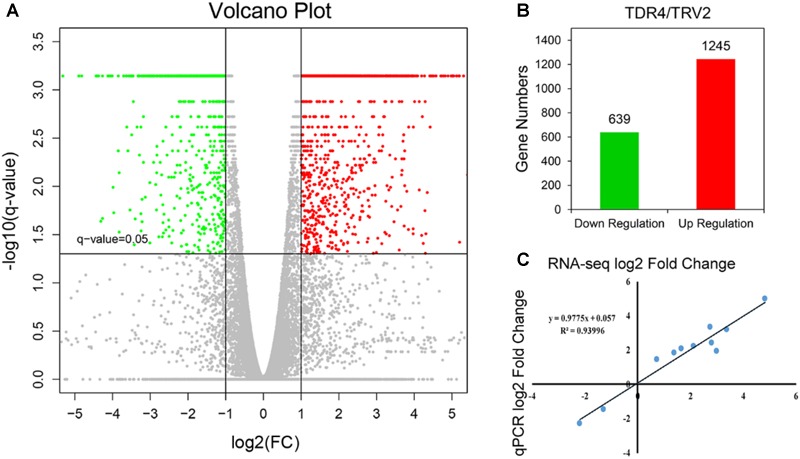
Global overview of DEGs between *TDR4*-silenced and control tomato fruit. **(A)** Volcano plot of DEGs. Spots above the threshold line (Q-value = 0.05) indicate significant DEGs. Genes for which expression in *TDR4*-silenced fruit was less than half that in control fruit, with a Q-value < 0.05, are shown in green, while those for which expression in *TRD4*-silenced fruit was more than two fold that in the control group are shown in red. Genes in gray were neither up- nor downregulated. **(B)** Number of down- (639) and upregulated (1245) genes. **(C)** Expression levels of 11 genes as determined by qRT-PCR are closely correlated with those according to RNA-seq.

**FIGURE 3:**
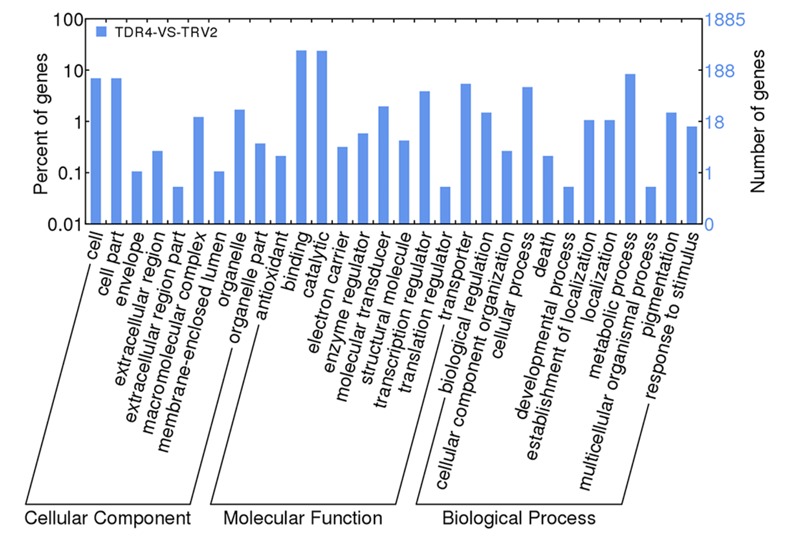
GO analysis of DEGs between *TDR4*-silenced and control tomato fruit according to WEGO.

**FIGURE 4:**
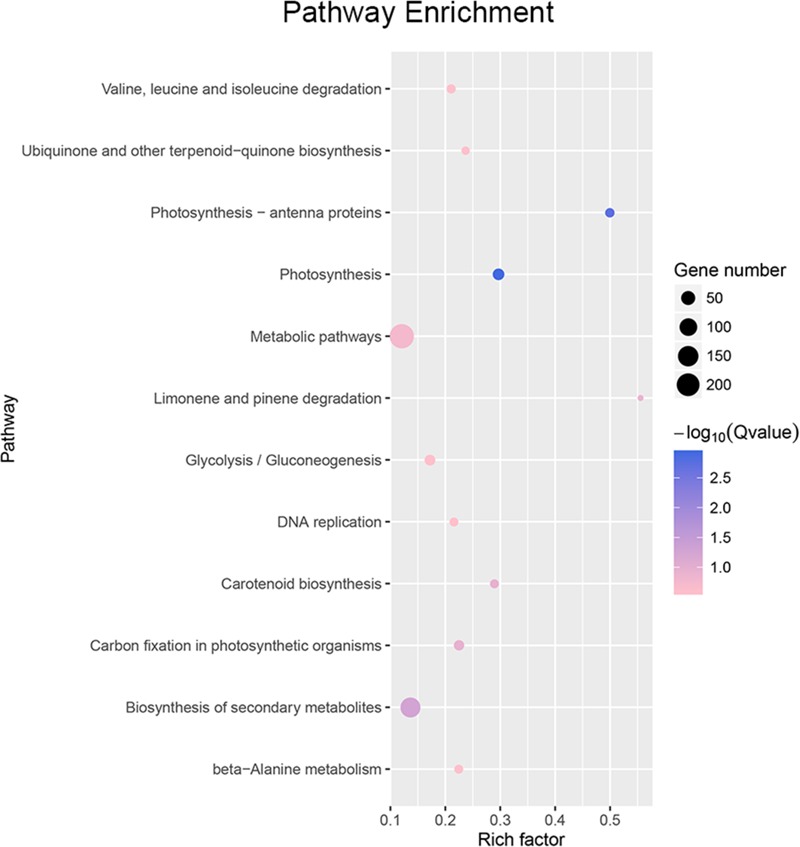
Pathway enrichment analysis of DEGs between *TDR4*-silenced and control tomato fruit.

**FIGURE 5:**
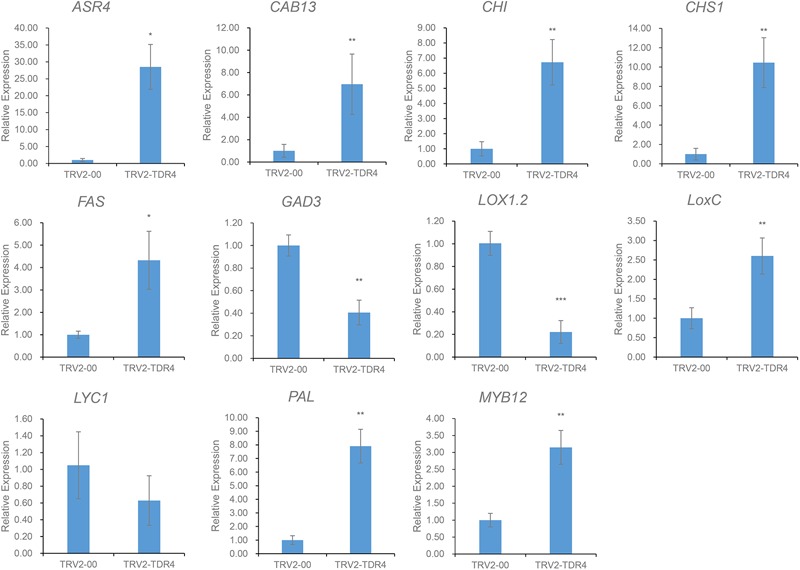
Expression levels of 11 genes according to qRT-PCR. Error bars indicate the standard deviation of three biological replicates. Asterisks indicate significant differences as determined by Student’s *t*-tests (^∗^*P* < 0.05, ^∗∗^*P* < 0.01, and ^∗∗∗^*P* < 0.001).

Using cutoff criteria with an expression ratio of ≥2 and *P* < 0.05 between TDR4-silencd and control tissues, analysis of DEGs revealed that 1245 genes were upregulated while 639 genes were downregulated in the *TDR4*-silenced fruits compared with that in control fruit (Figure 2). To provide an overview of the role of *TDR4*, we evaluated the DEGs using GO and KEGG pathway enrichment analyses. GO analysis indicated that *TDR4* silencing affected multiple metabolic pathways including 9 cellular component GO terms, with cell and cell part being the most enriched terms; 10 molecular function terms, with binding and catalytic being the most enriched; and 11 biological process terms, with metabolic process being the most enriched (Figure 3). KEGG pathway enrichment analysis showed that *TDR4* was involved in photosynthesis and the biosynthesis of secondary metabolites (Figure 4). Eleven genes related to fruit ripening and nutrient metabolism were selected for qRT-PCR validation of the RNA-seq data. The RT-qPCR results were consistent with the sequencing data, indicating the reliability of the sequencing results (Figure 5).

### Analysis of Metabolites in Tomato Fruit Samples

**FIGURE 6:**
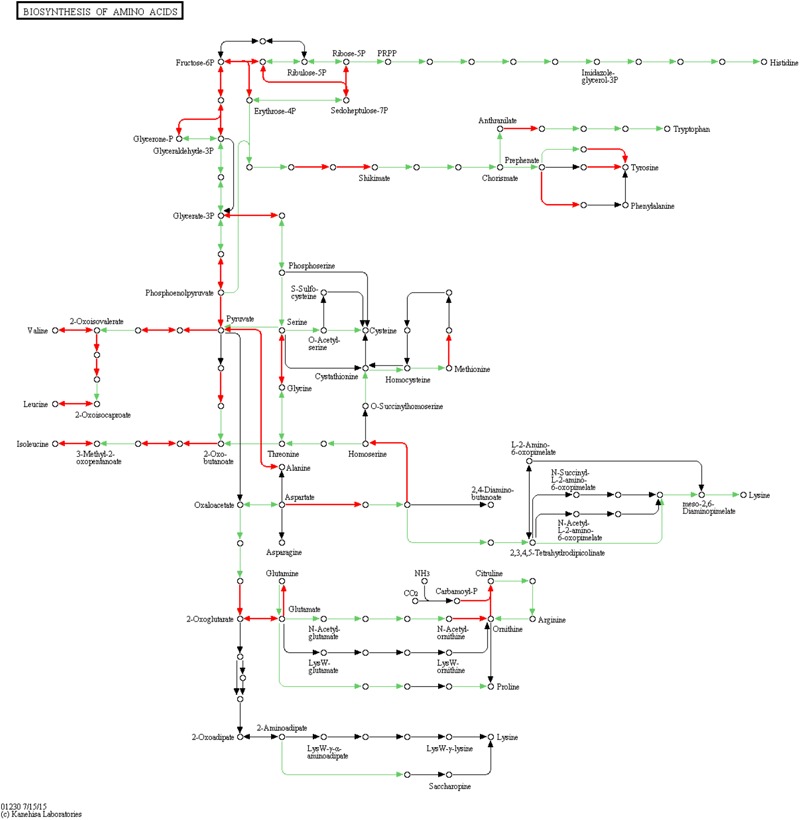
Schematic diagram of amino acid biosynthesis pathway. Diagram constructed using KEGG pathway analysis. Circles represent metabolites, and arrows indicate steps. Red arrows indicate steps catalyzed by DEGs.

To examine metabolic changes in the *TDR4* silenced fruit, LC-MS/MS metabolite analysis was performed in *TDR4*-silenced and control tomato fruits. The result indicates that 50 metabolites were identified in *TDR4*-silenced tomato fruit. According to a two-way analysis of variance (ANOVA), 17 of these metabolites significantly differed in abundance between control (TRV2-00) and *TDR4* silenced (TRV2-*TDR4)* fruit tissues (Table 2). All differential metabolites were further classified into four groups: amino acids, organic acids, phenolics, and solanum alkaloids. Possible pathways for each metabolite were determined by searching the KEGG database (Figure 6). These results suggest that the silencing of *TDR4* altered the tomato fruit metabolism.

**Table 2:** Relative quantitation of metabolites in *TDR4*-silenced and control tomato fruit.

Analytes	Ratio (TDR4-silenced/ control)	*P*-value^a^	Pathway
L-Tyrosine	0.32	3.28E-02	Biosynthesis of amino acids
L-Phenylalanine	0.25	4.69E-02	Biosynthesis of amino acids
L-Glutamic acid	0.53	8.29E-04	Biosynthesis of amino acids
Glutathione	0.53	2.50E-08	Glutathione metabolism
Eriodictyol chalcone	3.06	1.94E-03	Flavonoid biosynthesis
5-Caffeoylquinic acid	0.39	3.39E-04	-
α-Tomatine	7.69	1.32E-14	-
Benzoic acid	3.89	4.00E-09	Phenylalanine metabolism
Malic acid	1.30	1.28E-06	Citrate cycle (TCA cycle)
Citric acid	0.90	2.38E-04	Citrate cycle (TCA cycle)
Kaempferol-3-rutinoside	0.41	8.87E-03	-
4-Aminobenzamide, allopurinol, hypoxanthine	0.73	2.41E-02	-
Quercetin-hexose-deoxyhexose, -pentose	0.72	2.88E-02	-
C_11_H_23_O_12_P	0.43	2.51E-03	-
C_16_H_20_O_10_	1.52	1.88E-02	-
C_34_H_46_O_14_	1.38	3.40E-03	-
C_14_H_20_N_2_O_3_	0.27	2.04E-02	-

*^*a*^*P*values calculated using Student’s *t*-tests.*

### Silencing of *TDR4* Alters Fruit Metabolism

Amino acids are primary metabolites that contribute to the flavor and nutritional value of tomato fruits. In *TDR4*-silenced fruit, levels of three amino acids were significantly reduced, including L-tyrosine (–65%), L-phenylalanine (–75%), and L-glutamic acid (–47%) (Table 2). KEGG pathway analysis revealed that the metabolic pathway of aromatic amino acids, such as phenylalanine and tyrosine, biosynthesis, and glutamate metabolism were also altered in *TDR4*-silenced fruits compared to control fruit (Figure 4), indicating that *TDR4* gene plays a role in the accumulation of certain amino acids during tomato fruit ripening, which contributes to flavor formation of tomato fruit. Tomato fruit synthesizes flavor and nutrients during its ripening, and ripening process is regulated by ripening-related TFs, such as *AP2a* and *Rin* TFs, so these TFs may also participate in the regulation of flavor synthesis. The majority of the amino acids (15 out of 22) were present at significantly lower levels in the AP2i fruits than in the wild type, and the most dramatic reductions were in β-Ala, Ile, Met, Phe, and Trp (Karlova et al., 2011). Rin protein can target the promoter of *TomloxC* and *ADH2* genes, which encode lipoxygenase (LOX) and alcohol dehydrogenase, respectively, and are critical for the production of characteristic tomato aromas derived from LOX pathway (Qin et al., 2012).

Phenylalanine is an important precursor of many aroma volatiles and flavonoids. For example, 2-phenylacetaldehyde and 2-phenylethanol are derived from phenylalanine (Tieman et al., 2006); both of these have pleasant fruity, floral odors, and important biological functions in plants (Knudsen et al., 1993). They attract mammals and other seed dispersers and exhibit antimicrobial properties (Goff and Klee, 2006).

RNA-seq result show that two previously unreported genes (*Solyc11g066890* and *Solyc06g050630*), that encode prephenate dehydratase proteins, were significantly upregulated in *TDR4*-silenced fruit (Supplementary Table S3). These are probably involved in the first step of the sub-pathway that synthesizes L-phenylalanine or L-tyrosine, respectively, from L-arogenate. A gene (*Solyc10g038080*) encoding a shikimate dehydrogenase appears to be downregulated in *TDR4*-silenced fruits (Supplementary Table S2), which may contribute to a reduction in shikimate, an important precursor of L-phenylalanine and L-tyrosine.

Benzoic acid, synthesized from trans-cinnamate, is hypothesized to be the functional group in salicylic acid, and its derivatives are assumed to be involved in inducing stress tolerance in plants (Senaratna et al., 2003). In our study, the content of benzoic acid increased (3.89-fold) (Table 2) in *TDR4*-silenced fruit, along with the expression of two phenylalanine ammonia-lyase (PAL) genes. PALs are key enzymes in plant metabolism, catalyzing the first step of the sub-pathway that synthesizes trans-cinnamate from L-phenylalanine. One of the two upregulated genes is *PAL5* (Supplementary Table S2), which is strongly expressed in old leaves and flowers and may function in response to biotic and abiotic stresses (Guo and Wang, 2009; Puthoff et al., 2010). Our findings suggest that the *TDR4* gene negatively regulates the expression of *PALs* to inhibit the synthesis of benzoic acid in tomato fruit.

Glutamic acid is the most abundant amino acid in the diet, and a high level of free glutamate in some foods results in an umami taste (e.g., tomatoes, mushrooms, cheeses) (Bellisle, 1999). During tomato ripening, the glutamic acid content rises dramatically (Bemer et al., 2012). In our study, the expression levels of five related genes were significantly different in *TDR4*-silenced and control tomato fruit, and four of these were upregulated, including glutamate dehydrogenase (*GDH1*) (1.11-fold), glutamine synthetase (*GS*) (6.04-fold), glutamate decarboxylase 1 (*GAD3*) (2.20-fold), and a gene encoding isocitrate dehydrogenase (1.99-fold) (Supplementary Table S2). GDH1, which acts in the mitochondria, catalyzes the reversible amination of 2-oxoglutarate to L-glutamic acid (Ferraro et al., 2015). GS is a chloroplast glutamine synthetase that assimilates ammonia into glutamine (Perez-Rodriguez and Valpuesta, 1996), which is a metabolic intermediate in the synthesis of other nitrogen-containing compounds in plants (Perez-Rodriguez and Valpuesta, 1996). GAD3 converts L-glutamic acid to γ-aminobutanoic acid (GABA). The increased transcript abundances of these genes indicate the acceleration of glutamate metabolism in *TDR4*-silenced fruit.

Glutathione (GSH) is a tripeptide (γ-glutamylcysteinylglycine) that exists in a broad range of organisms, from bacteria to humans (Frendo et al., 2013). In humans, GSH plays an important role in the metabolism and detoxification of cytotoxic and carcinogenic compounds and reactive oxygen species (ROS) (Knapen et al., 1999). In plants, GSH is crucial for plant development and the plant response to the abiotic and biotic environment, and it is also involved in the detoxification of xenobiotics (Frendo et al., 2013; Yu et al., 2013). In our analysis of tomato fruit, *TDR4* silencing resulted in a significant reduction in glutathione (–47%) (Table 2). KEGG pathway analysis showed that the *GST* gene, encoding glutathione S-transferase, was upregulated, which would promote the conversion of glutathione to glutamate.

Eriodictyol chalcone is a type of flavonoid. It has been reported that eriodictyol chalcone accumulates predominantly in the tomato peel and exhibits the highest accumulation at the breaker stage, gradually decreasing during ripening (Iijima et al., 2008). Tomato *SlAN11* regulates flavonoid biosynthesis (Gao et al., 2018a). In our study, the content of eriodictyol chalcone was significantly increased in *TDR4*-silenced fruit (3.06-fold) (Table 2), compared to that in controls. KEGG pathway analysis showed that flavonoid biosynthesis was altered in *TDR4*-silenced fruit, which was consistent with the observation that chalcone synthase 1 (*CHS1*) and chalcone synthase 2 (*CHS2*) were upregulated (Supplementary Table S2).

5-Caffeoylquinic acid (chlorogenic acid) is one of the most abundant and widespread soluble phenolics among vascular plants (Mahesh et al., 2007). Evidence suggests that it can protect plant cells against oxidative stress, and plays a role in resistance to phytopathogens (Mahesh et al., 2007). The existence of another route involving the direct 3′-hydroxylation of *p*-coumaryol quinic acid was first suggested in carrot cell cultures, and studies of the impact of the expression of the hydroxycinnamoyl quinic acid gene in tobacco and tomato plants demonstrated that this route may be predominant in the Solanaceae family (Niggeweg et al., 2004). Analysis of *TDR4*-silenced tomato fruit revealed a significant reduction in 5-caffeoylquinic acid levels (–61%) (Table 2).

α-Tomatine is an anti-nutritional factor for humans (Friedman, 2013). In tomato, α-tomatine is present at high concentrations during the mature green (MG) stage and dramatically decreases during fruit ripening (Mintz-oron et al., 2008). In the present study, levels of α-tomatine were significantly elevated in *TDR4*-silenced fruit (7.69-fold) (Table 2). At the same time, the expression level of *GAME11* was increased in *TDR4*-silenced fruit (Supplementary Table S3). It was reported that using VIGS technology to silence *GAME11*a putative dioxygenase in the cluster resulted in a significant reduction in α-tomatine levels and accumulation of several cholestanol-type steroidal saponins in tomato leaves (Itkin et al., 2013), which was consistent with our results. Putatively, GAME11 catalyzes the closure of the E-ring of 22,26-dihydroxycholesterol to form the furostanol-type aglycone (Itkin et al., 2013). In summary, the silencing of *TDR4* promotes α-tomatine biosynthesis by enhancing the expression of *GAME11* and TDR4 is a negative regulator of *GAM11* gene.

## Conclusion

The silencing of *TDR4* using VIGS resulted in a non-ripening phenotype with an orange pericarp. RNA-seq analysis of *TDR4*-silenced fruit showed the altered expression of genes involved in various metabolic pathways. Analysis of metabolites by LC-MS/MS showed reductions in several amino acids as well as the accumulation of α-tomatine in *TDR4*-silenced fruit. These results suggest that *TDR4* regulates the accumulation of nutrients and flavor in tomato fruit via the transcriptional regulation of target genes.

## Author Contributions

XZ designed the experiments and wrote the manuscript. XY and SC did the experiments. D-QF analyzed the data. C-ZJ revised the manuscript.

## Conflict of Interest Statement

The authors declare that the research was conducted in the absence of any commercial or financial relationships that could be construed as a potential conflict of interest.

## Footnotes

1 https://pypi.python.org/pypi/cutadapt/

2 http://hannonlab.cshl.edu/fastx_toolkit/download.html

3 ftp://ftp.sgn.cornell.edu/tomato_genome

4 http://ccb.jhu.edu/software/tophat/index.shtml

5 http://cole-trapnell-lab.github.io/cufflinks/

6 https://www.ncbi.nlm.nih.gov/Traces/study/?acc=SRP201254

7 http://wego.genomics.org.cn/

8 http://kobas.cbi.pku.edu.cn/download.php

## Supplementary Material

TABLE S1 Oligonucleotide primers used in the study.

TABLE S2 Representative DEGs in *TDR4-*silenced tomato fruits.

TABLE S3 The DEGs in *TDR4-*silenced tomato fruits.
