# Supplementary material for: Metabolomic and Transcriptomic Analyses Reveal That a MADS-Box Transcription Factor TDR4 Regulates Tomato Fruit Quality
Source: Front Plant Sci. 2019 Jun 19;10:792. doi: 10.3389/fpls.2019.00792 (PMC6593160; doi:10.3389/fpls.2019.00792)
Supplement: TABLE S1 — Oligonucleotide primers used in the study. [file Table_1.DOCX]

**Table S1. Oligonucleotide primers used in the study.**

| **Primer** | **Sequence** |
| --- | --- |
| qPCR-LYC1-F | AGTTCTTCTGCTTCGGTATGG |
| qPCR-LYC1-R | GAGACGATAAGAAGCCATGCC |
| qPCR-FAS-F | CTTCGATTCGTCCACCAGAG |
| qPCR-FAS-R | TCCAGCTTGAGTCCAAAGTG |
| qPCR-ASR4-F | TTTGCTTTGCATGAGAAGCAC |
| qPCR-ASR4-R | TTCTTCTTTCCCTCAGCTTCC |
| qPCR-GAD3-F | CTTGGGCTATGAGGGATATCG |
| qPCR-GAD3-R | AGACCTCGAATTCGTTGTGGA |
| qPCR-MYB12-F | GGGTAACAGATGGTCTCTTATAGC |
| qPCR-MYB12-R | GGCTTTAGGTAACTTCTCATCG |
| qPCR-LOX1.2-F | TCATTAGCCACTGGTTGAATACAC |
| qPCR-LOX1.2-R | GATGAAGCACACTTAGATGCCT |
| qPCR-LoxC-F | AGCTATGGAGGCTACTTTCCA |
| qPCR-LoxC-R | ATTCAAGAACCACTCCCATTCC |
| qPCR-CHS1-F | AGGGGTCGAAAGACCTTTAT |
| qPCR-CHS1-R | TCCCTAGAGGTTGAAATGCT |
| qPCR-CAB13-F | CTCTTGAGGTTATCCATGGGAG |
| qPCR-CAB13-R | TTGCCCAAATAGTCCAGCC |
| qPCR-PAL-F | TTTGGTGCTACATCTCATAGGA |
| qPCR-PAL-R | TGACATGACTCAGTACCATTACC |
| qPCR-CHI-F | GTGCAAAGATATCGTTCGCGA |
| qPCR-CHI-R | CCCTTGTTCCACCTAAGTACCA |
| qPCR-TDR4-F | TCGAAGAAGGTGAAGGAGAGG |
| qPCR-TDR4-R | CTTGCTGCTGTGAAGAACTACC |
| qPCR-FUL2-F | TCAAAGCAGGTTAAGGAAAGG |
| qPCR-FUL2-R | CTTCTACTTCTCCATTGTCTCC |
| qPCR-Actin-F | CAGCAGATGTGGATCTCAAA |
| qPCR-Actin-R | CTGTGGACAATGGAAGGAC |
| VIGS-TDR4-F | ATCCGAGGATCCTACTCATT TGCTGAGAAACA |
| VIGS-TDR4-R | GTACTGGAATTCAAATGAAGATGAGTTGATTT |
