# Supplementary material for: Metabolomic and Transcriptomic Analyses Reveal That a MADS-Box Transcription Factor TDR4 Regulates Tomato Fruit Quality
Source: Front Plant Sci. 2019 Jun 19;10:792. doi: 10.3389/fpls.2019.00792 (PMC6593160; doi:10.3389/fpls.2019.00792)
Supplement: TABLE S2 — Representative DEGs in TDR4-silenced tomato fruits. [file Table_2.DOCX]

**Table S2. Representative DEGs in *TDR4-*silenced tomato fruits.**

| Accession | Annotation | Ratio |
| --- | --- | --- |
| **Photosynthesis** |  |  |
| Solyc05g016120 | Photosystem Q | -1.16483 |
| Solyc09g065910 | Photosystem II reaction center W protein | 2.13772 |
| Solyc07g066150 | Photosystem I reaction center subunit V | 1.9728 |
| Solyc06g082950 | Photosystem I reaction center subunit XI | 2.57831 |
| Solyc07g044860 | Oxygen-evolving enhancer protein 2, chloroplastic | 1.00219 |
| Solyc01g007520 | Photosystem II reaction center protein H | -1.51314 |
| Solyc12g056830 | ATP synthase delta subunit | 1.66037 |
| Solyc02g080540 | ATP synthase gamma chain | 3.3373 |
| Solyc04g082010 | Plastocyanin | 1.5253 |
| Solyc06g054260 | Photosystem I reaction center subunit II | 1.64226 |
| Solyc08g006930 | Photosystem I reaction center subunit X psaK | 5.01449 |
| Solyc09g063130 | Photosystem I reaction center subunit IV A | 3.2736 |
| Solyc06g074200 | Sex-linked protein 9 | 5.59384 |
| Solyc06g084050 | Photosystem II reaction center W protein | 2.9189 |
| Solyc10g044520 | Ferredoxin I | 1.40788 |
| Solyc02g090030 | Oxygen-evolving enhancer protein 1 of photosystem II | 2.75642 |
| Solyc02g065400 | Oxygen-evolving enhancer protein 1 of photosystem II | 1.47955 |
| Solyc05g007780 | Oxygen evolving enhancer protein 3 | 3.13671 |
| Solyc06g083680 | Photosystem I reaction center subunit IV A | 1.9259 |
| Solyc02g079950 | Oxygen-evolving enhancer protein 3 | 3.53911 |
| Solyc08g013670 | Photosystem I reaction center subunit | 6.88854 |
| Solyc06g082940 | Photosystem I reaction center subunit XI | 3.79021 |
| Solyc06g060340 | Chloroplast photosystem II-associated protein | 2.41804 |
| Solyc07g054290 | Photosystem II family protein | 1.135 |
| Solyc07g066310 | photosystem II polypeptide | 1.56328 |
| Solyc02g069450 | Photosystem I reaction center subunit III | 1.80695 |
| **Photosynthesis - antenna proteins** | |  |
| Solyc06g063370 | Chlorophyll a-b binding protein 1A | 2.3913 |
| Solyc03g005760 | Chlorophyll a-b binding protein 3C-like | 5.18027 |
| Solyc02g071030 | Chlorophyll a/b binding protein | 4.23903 |
| Solyc12g009200 | Chlorophyll a-b binding protein | 3.64519 |
| Solyc02g070940 | Chlorophyll a/b binding protein | 1.62985 |
| Solyc07g047850 | Chlorophyll a-b binding protein 4 | 2.68648 |
| Solyc09g014520 | Chlorophyll a-b binding protein 6A | 1.07959 |
| Solyc10g006230 | Chlorophyll a-b binding protein 7 | 3.80415 |
| Solyc01g105030 | Chlorophyll a-b binding protein | 3.67081 |
| Solyc07g063600 | Chlorophyll a-b binding protein 13 | 2.36742 |
| Solyc10g007690 | Chlorophyll a-b binding protein 8 | 2.7508 |
| Solyc01g105050 | Chlorophyll a-b binding protein | 3.53682 |
| **Carotenoid biosynthesis** | |  |
| Solyc12g056600 | Short chain alcohol dehydrogenase | 3.52924 |
| Solyc04g078900 | Cytochrome P450 | -2.31909 |
| Solyc04g050930 | Violaxanthin de-epoxidase | 1.71859 |
| Solyc03g007960 | Beta-carotene hydroxylase 2 | -1.14677 |
| Solyc04g040190 | Lycopene beta-cyclase 1 | 1.39797 |
| Solyc11g071600 | Aldehyde oxidase | 2.97829 |
| Solyc01g009230 | Xanthine dehydrogenase/oxidase | 1.31871 |
| Solyc08g016720 | 9-cis-epoxycarotenoid dioxygenase 5 | 1.53625 |
| Solyc08g075320 | Cytochrome P450 | 1.72483 |
| Solyc07g056570 | 9-cis-epoxycarotenoid dioxygenase | 1.17473 |
| Solyc02g090890 | Zeaxanthin epoxidase, chloroplastic | 1.39868 |
| **Glycolysis / Gluconeogenesis** | |  |
| Solyc10g085550 | Enolase | 1.74368 |
| Solyc04g074530 | Alcohol dehydrogenase 1 | 2.51941 |
| Solyc02g086970 | Aldehyde dehydrogenase 1 | -1.6375 |
| Solyc02g084640 | Aldehyde dehydrogenase | 1.24434 |
| Solyc03g114150 | Aldehyde dehydrogenase | 1.09506 |
| Solyc07g005390 | Aldehyde dehydrogenase | 2.75369 |
| Solyc01g110360 | Fructose-bisphosphate aldolase | 2.81826 |
| Solyc12g094500 | Alcohol dehydrogenase 1 | -1.07349 |
| Solyc10g086730 | Fructose-1 6-bisphosphatase class 1 | 2.94589 |
| Solyc08g066100 | Phosphofructokinase family protein | 2.20603 |
| Solyc09g011810 | Fructose-1 6-bisphosphatase class 1 | 3.81826 |
| Solyc02g062340 | Fructose-bisphosphate aldolase | 5.308 |
| Solyc06g072160 | Alcohol dehydrogenase 1 | 1.63427 |
| Solyc10g083720 | Pyruvate kinase | -1.526 |
| Solyc02g087780 | Aldose-1-epimerase-like protein | 2.1607 |
| Solyc05g005700 | Aldehyde dehydrogenase 1 | 3.7302 |
| Solyc03g006870 | Phosphoglucomutase | -1.1801 |
| Solyc10g076510 | Pyruvate decarboxylase | -1.42536 |
| Solyc03g114500 | Enolase | 1.35401 |
| Solyc05g053100 | Dihydrolipoyl dehydrogenase | 2.55412 |
| Solyc02g084440 | Fructose-bisphosphate aldolase | 1.00555 |
| **Flavonoid biosynthesis** | |  |
| Solyc09g091510 | Chalcone synthase | 3.16147 |
| Solyc05g053550 | Chalcone synthase | 2.46379 |
| Solyc10g050160 | Caffeoyl-CoA 3-O-methyltransferase | -1.72122 |
| Solyc03g115220 | Cytochrome P450 | 3.23307 |
| Solyc11g013110 | Anthocyanidin synthase | 2.80004 |
| Solyc02g083860 | Flavanone 3-hydroxylase | 3.30193 |
| Solyc10g078240 | Cytochrome P450 | 1.19459 |
| **Glutathione metabolism** | |  |
| Solyc09g063150 | Glutathione S-transferase | 3.12483 |
| Solyc04g026030 | Spermidine synthase | -1.19297 |
| Solyc04g051350 | Ribonucleoside-diphosphate reductase | -1.65326 |
| Solyc01g099590 | Glutathione-S-transferase | -1.21597 |
| Solyc01g005560 | Isocitrate dehydrogenase | 1.98651 |
| Solyc02g094160 | Ribonucleoside-diphosphate reductase small chain | -2.36575 |
| Solyc04g009530 | Glutathione S-transferase | 1.88936 |
| Solyc09g011630 | Glutathione S-transferase-like protein | -2.18394 |
| Solyc07g056490 | Glutathione transferase | 1.30082 |
| Solyc07g045540 | Glucose-6-phosphate 1-dehydrogenase | 1.04212 |
| Solyc09g011560 | Glutathione S-transferase-like protein | 1.56316 |
| Solyc01g081250 | Glutathione-S-transferase | -2.76152 |
| Solyc12g011320 | Glutathione S-transferase | 1.34979 |
| **Valine, leucine and isoleucine degradation** | |  |
| Solyc02g086970 | Aldehyde dehydrogenase 1 | -1.6375 |
| Solyc01g108030 | Methylcrotonoyl-CoA carboxylase beta subunit | 1.15992 |
| Solyc02g084640 | Aldehyde dehydrogenase | 1.24434 |
| Solyc04g063350 | 3-methyl-2-oxobutanoate dehydrogenase | 2.788 |
| Solyc05g056480 | Pyruvate dehydrogenase E1 component alpha subunit | 1.84437 |
| Solyc12g088220 | Branched-chain-amino-acid aminotransferase | -1.80294 |
| Solyc12g011160 | Enoyl-CoA-hydratase | 1.09146 |
| Solyc03g114150 | Aldehyde dehydrogenase | 1.09506 |
| Solyc05g032680 | Enoyl-CoA-hydratase | 2.25037 |
| Solyc05g005700 | Aldehyde dehydrogenase 1 | 3.7302 |
| Solyc05g053100 | Dihydrolipoyl dehydrogenase | 2.55412 |
| **beta-Alanine metabolism** | |  |
| Solyc01g087590 | Polyamine oxidase | -2.5872 |
| Solyc02g086970 | Aldehyde dehydrogenase 1 | -1.6375 |
| Solyc04g026030 | Spermidine synthase | -1.19297 |
| Solyc02g084640 | Aldehyde dehydrogenase | 1.24434 |
| Solyc03g098240 | Glutamate decarboxylase | 2.20392 |
| Solyc12g011160 | Enoyl-CoA-hydratase | 1.09146 |
| Solyc03g114150 | Aldehyde dehydrogenase | 1.09506 |
| Solyc01g005000 | Glutamate decarboxylase | -1.47865 |
| Solyc05g005700 | Aldehyde dehydrogenase 1 | 3.7302 |
| Solyc11g064920 | Dihydropyrimidinase | 1.22719 |
| Solyc05g032680 | Enoyl-CoA-hydratase | 2.25037 |
| **Carbon fixation in photosynthetic organisms** | |  |
| Solyc04g009030 | Glyceraldehyde-3-phosphate dehydrogenase | 1.96319 |
| Solyc05g013380 | Alanine aminotransferase 2 | 1.32861 |
| Solyc04g082630 | Glyceraldehyde-3-phosphate dehydrogenase B | 3.95103 |
| Solyc02g020940 | Glyceraldehyde-3-phosphate dehydrogenase | 4.27187 |
| Solyc01g110360 | Fructose-bisphosphate aldolase | 2.81826 |
| Solyc04g006970 | Phosphoenolpyruvate carboxylase | 1.24728 |
| Solyc08g066360 | Malic enzyme | 1.7702 |
| Solyc10g086730 | Fructose-1 6-bisphosphatase class 1 | 2.94589 |
| Solyc09g011810 | Fructose-1 6-bisphosphatase class 1 | 3.81826 |
| Solyc02g062340 | Fructose-bisphosphate aldolase | 5.308 |
| Solyc02g084440 | Fructose-bisphosphate aldolase | 1.00555 |
| Solyc11g007990 | Malate dehydrogenase | 1.45006 |
| Solyc01g007330 | Ribulose bisphosphate carboxylase large chain | -2.16626 |
| Solyc10g018300 | Transketolase 1 | 1.85134 |
| Solyc05g052600 | Fructose-1 6-bisphosphatase class 1 | 1.51824 |
| Solyc08g076220 | Phosphoribulokinase/uridine kinase | 2.92798 |
| Solyc03g034220 | Ribulose bisphosphate carboxylase small chain | 3.27872 |
| **DNA replication** |  |  |
| Solyc07g005020 | DNA replication licensing factor | -1.638 |
| Solyc08g082200 | DNA primase | -2.77049 |
| Solyc02g070780 | DNA replication licensing factor MCM3 | -2.15364 |
| Solyc06g076660 | Proliferating cell nuclear antigen | -1.43411 |
| Solyc11g040120 | DNA replication licensing factor | -1.1931 |
| Solyc05g014540 | DNA polymerase alpha subunit B family | -2.18783 |
| Solyc01g110130 | DNA replication licensing factor | -1.75622 |
| Solyc11g067110 | DNA polymerase | -1.06912 |
| Solyc04g045530 | DNA primase large subunit | -1.41178 |
| Solyc02g082180 | DNA replication licensing factor | -1.22266 |
| Solyc07g018300 | Single-stranded DNA binding protein p30 subunit | -1.77651 |
| **Fatty acid degradation** | |  |
| Solyc04g074530 | Alcohol dehydrogenase 1 | 2.51941 |
| Solyc06g072160 | Alcohol dehydrogenase 1 | 1.63427 |
| Solyc02g086970 | Aldehyde dehydrogenase 1 | -1.6375 |
| Solyc02g084640 | Aldehyde dehydrogenase | 1.24434 |
| Solyc12g094500 | Alcohol dehydrogenase 1 | -1.07349 |
| Solyc01g079240 | Long-chain-fatty-acid--CoA ligase family protein | 2.75805 |
| Solyc03g114150 | Aldehyde dehydrogenase | 1.09506 |
| Solyc10g085200 | Acyl-CoA dehydrogenase | 2.49535 |
| Solyc01g095750 | Long-chain-fatty-acid-CoA ligase | 4.28992 |
| Solyc05g005700 | Aldehyde dehydrogenase 1 | 3.7302 |
| **Ubiquinone and other terpenoid-quinone biosynthesis** | |  |
| Solyc03g117870 | 4-coumarate CoA ligase | -1.21295 |
| Solyc04g005180 | 2-oxoglutarate decarboxylase/ hydro-lyase/ magnesium ion binding protein | 1.68989 |
| Solyc08g068570 | Tocopherol cyclase | -1.16203 |
| Solyc02g069920 | Long-chain-fatty-acid CoA ligase | 2.69257 |
| Solyc07g053720 | Tyrosine aminotransferase-like protein | -1.7101 |
| Solyc08g076300 | 4-coumarate-coa ligase | -1.55625 |
| Solyc05g005180 | Naphthoate synthase | 1.71497 |
| Solyc07g017770 | Homogentisate phytyltransferase | -1.17409 |
| Solyc03g097030 | 4-coumarate CoA ligase | 2.92988 |
